# How Does COVID-19 Risk Perception Affect Sense of Control? The Roles of Death Anxiety and Confucian Coping

**DOI:** 10.3390/ijerph20032299

**Published:** 2023-01-28

**Authors:** Lianqiong Huang, Yubo Hou, Zhaoyang Sun, Qi Wang

**Affiliations:** 1 School of Psychological and Cognitive Sciences, Beijing Key Laboratory of Behavior and Mental Health, Peking University, Beijing 100871, China; 2 College of Human Ecology, Cornell University, Ithaca, NY 14853, USA

**Keywords:** COVID-19 risk perception, sense of control, death anxiety, Confucian coping

## Abstract

This research examined the impact of COVID-19 risk perception on sense of control, testing the hypotheses that COVID-19 risk perception would reduce sense of control and that this effect would be mediated by death anxiety and moderated by Confucian coping. A series of six studies were conducted with Chinese participants (*N* = 2202) and employed different research designs in lab and real-life settings. Across the studies, we found that the perceived risk of COVID-19 impaired sense of control. Studies 3a to 5 further revealed that death anxiety mediated the adverse effect of COVID-19 risk perception on sense of control, and Studies 4 to 5 revealed that Confucian coping strategies alleviated the adverse effect of COVID-19 risk perception on sense of control. These findings shed new light on the psychological impact of risk perception in times of crisis and identify mitigating factors and boundary conditions.

## 1. Introduction

Sense of control is the belief that one can predict, influence, and steer current or future events in life [1]. Having a high level of sense of control has been shown to be beneficial for an individual’s psychosocial adjustment [2,3], physical health [4], mental health [5], and life satisfaction [6]. Conversely, the lack of a sense of control is linked to a variety of physical and emotional problems such as health risks, anxiety, and depression [7,8]. A long-term loss of sense of control can also lead to learned helplessness [9,10]. Existing studies suggest that individuals’ sense of control is influenced by the sociocultural context [11], demographic factors [12,13], and life events [14]. However, little is known about the antecedents and boundary conditions that affect the sense of control, a question that is of paramount importance in times of crisis.

At the end of 2019, the sudden outbreak of the COVID-19 pandemic took place. As a major public health emergency, the rapid spread of Coronavirus has drastically changed people’s daily lives around the world [15]. Even to this date, it is still unclear how long the pandemic will last. The pandemic has created a sense of powerlessness in individuals and undermined their sense of control [16], resulting in many negative psychological reactions such as fear, anger, frustration, helplessness, loneliness, and intense uncertainty [17,18,19]. Against this backdrop, the present research examines the impact of COVID-19 risk perception on sense of control and further investigates the roles of death anxiety and Confucian coping in the relation between risk perception and sense of control.

### 1.1. COVID-19 Risk Perception and Sense of Control

Risk perception refers to individuals’ subjective assessment of hazardous factors in the external environment that may threaten their health and well-being [20,21,22]. It may be associated with the severity of a crisis [23] and can have significant impacts on individuals’ attitudes [24], behaviors [25], and psychological well-being [26,27].

The outbreak of the COVID-19 pandemic around the globe triggered an unprecedented crisis, which stagnated economic growth, interrupted social orders, and changed every aspect of daily life [15]. This pandemic is characterized by high infectivity, widespread areas, a long duration, and serious consequences of infection [28]. To reduce infection, governments and authorities around the world have implemented a series of preventative measures, such as mandatory isolation, compulsory mask wearing, prohibition of mass gatherings, travel restrictions, and remote schooling [29,30,31]. Although these restrictions have helped to alleviate the pandemic, they are also constant reminders of the severity of COVID-19 and the possibility of infection, which may cause people to continuously perceive the risk of COVID-19.

Importantly, perceiving risks and threats in the environment, especially those beyond one’s control [32,33,34], may result in a reduced sense of control among individuals. Prior studies have suggested a negative relation between risk perception and sense of control, whereby individuals who perceive higher risk in their environment exhibit lower sense of control [5,35]. In the context of COVID-19, there is some evidence for a negative correlation between sense of control and the perceived threat of COVID-19 [36,37] and fear of COVID-19 [38]. The causal connection between COVID-19 risk perception and sense of control has yet to be established. Furthermore, if the perceived COVID-19 risk reduces one’s sense of control, death anxiety may play a role in underlying the effect.

### 1.2. The Mediating Role of Death Anxiety

Death anxiety reflects an emotional state of fear or dread caused by the threat of death, when the inevitability of death is remembered [39,40]. Studies have shown that diseases with high mortality tend to increase people’s anxiety about death [41,42]. COVID-19 is a highly contagious virus that has caused millions of deaths globally. As of January 2023, more than 6.72 million people have died worldwide, and there were over 664.87 million confirmed cases [43], with the numbers increasing daily. The increasing numbers of confirmed cases and deaths may not only remind people of the ongoing threat of COVID-19 to their lives and those of their loved ones, but also trigger their anxiety about death [44,45,46]. 

Studies have shown that individuals experience increased death anxiety following the increase in perceived risk of COVID-19 [42,47,48]. Moreover, a positive correlation has been identified between the perceived threat of COVID-19 and death anxiety [49], and between the perceived risk of COVID-19 and death distress [50]. Conceivably, death anxiety may, in turn, make individuals experience a loss of control [16]. Emerging research has suggested that death salience threatens individuals’ sense of control [51,52,53] and that COVID-19 related anxiety is negatively correlated with individuals’ sense of control over their health [54,55]. Taken together, it is possible that COVID-19 risk perception induces death anxiety, which in turn leads to a reduced sense of control. This possibility remains an empirical question to be examined.

### 1.3. The Moderating Role of Confucian Coping

Importantly, individuals are psychologically affected by COVID-19 to variable degrees, which suggests the importance of examining boundary conditions that may mitigate the negative impact of COVID-19. Individual factors such as self-compassion [49] and positivity [50] have been found to be helpful for alleviating the negative effect of COVID-19 on psychological functioning. Beliefs in the protective efficacy of mask-wearing [55] and social supports such as online counseling or mental health services [56] are also beneficial in protecting individuals’ sense of control in the context of COVID-19. However, little is known about how coping strategies help individuals deal with the risk of COVID-19 and maintain a sense of control.

Effective coping strategies often reflect not only individual characteristics but also cultural beliefs [57,58]. Confucian thinking has profoundly influenced Chinese society, shaping how individuals think, feel, behave, and cope with stressors in life [59,60,61]. In particular, Confucian thinking emphasizes the determination of fate, an appreciation for failures and setbacks, and a sense of responsibility in times of challenges, which may be used by Chinese individuals when confronting stressful situations like the COVID-19 pandemic. The tendency to use Confucian values to deal with stressful situations is referred to here as Confucian coping, which includes three dimensions: fate thinking, pro-setback thinking, and responsibility thinking [59].

Research has shown that in line with Confucian thinking, fate beliefs often serve as a common coping strategy for East Asians to appraise negative events as uncontrollable losses that must be accepted [61,62]. Moreover, fate beliefs are used by Asians to cope with death anxiety [63] and are positively associated with active coping among Chinese individuals [64]. Furthermore, taking responsibility and appreciating the value of setbacks, as active coping strategies, have been shown to be negatively associated with anxiety and depression and positively associated with psychological resilience and well-being among Chinese [65,66]. Thus, in the COVID-19 context, Confucian coping that encourages individuals to take active responsibility and be persistent, to view the negative situation as a matter of fate, and to consider setbacks beneficial for personal growth, may help individuals to reduce death anxiety and further protect their sense of control from the negative impact of COVID-19 risk perception. 

### 1.4. The Present Study

The present study examined the effect of COVID-19 risk perception on sense of control in Chinese individuals across six studies. All participants were recruited via the Chinese online platform Credamo (similar to M-Turk); they were from the general population and older than 18. For each study, we conducted a power analysis to determine the sample size and then recruited a larger and likely more representative sample, within our time and funding constraints. We hypothesized that COVID-19 risk perception would reduce sense of control and that this effect would be mediated by death anxiety and moderated by Confucian coping. Study 1 examined the correlation between COVID-19 risk perception and sense of control. Study 2 used an experimental manipulation (i.e., text priming) to examine the effect of COVID-19 risk perception on sense of control. Then, Study 3a tested the mediating role of death anxiety in the effect of COVID-19 risk perception on sense of control, using another experimental manipulation (i.e., video priming). Study 3b further manipulated death anxiety to replicate the findings of Study 3a. Study 4 examined the moderating role of pro-setback thinking, one aspect of Confucian coping, in the effect of COVID-19 risk perception on sense of control in a cross-sectional experiment. Study 5 tested all three aspects of Confucian coping in a moderated mediation model with three waves of data collection. Data collection took place between 1 December 2021 (the number of newly confirmed cases and deaths in China was 113 and 0, respectively) and 9 February 2022 (the number of newly confirmed cases and deaths in China was 110 and 0, respectively), when the COVID-19 infection status in China remained stable. 

The studies were approved by the Peking University IRB (Protocol #2022-02-15). All data and research materials are available at https://osf.io/r9us5/files/osfstorage?view_only=46c2cd66e94f42d4bee4adc9976ce646 (accessed on 1 December 2021).

### 1.5. Plan of Analysis

All analyses were performed with SPSS (Version 22), using statistical methods in line with the respective research designs and questions to be addressed. We conducted a correlation analysis in Study 1 to test the negative association between COVID-19 risk perception and sense of control. In Study 2, 3b, and 4, we performed independent sample *t*-tests for between-condition comparisons. In Study 3a, we conducted a MANOVA to test the main effect of risk perception on sense of control and death anxiety. In Study 4, we conducted a 2 × 2 ANOVA to test the interaction effect of risk perception and Confucian coping on sense of control. In Study 3a, 4, and 5, mediation analyses were performed to examine the mediating role of death anxiety in the relation of COVID-19 risk perception to sense of control. In Study 4 and 5, we conducted moderated mediation analyses to test the moderating role of Confucian coping.

Notably, we hypothesized based on the literature that death anxiety would be a mediator for the relation between risk perception and sense of control. However, an alternative possibility would be that sense of control served as a mediator for the relation between risk perception and death anxiety, such that higher risk perception might induce a lowered sense of control, which might in turn boost death anxiety. We therefore conducted additional mediation analyses to test this alternative pathway in Studies 3a, 4 and 5. The results showed that although the alternative mediation model was significant, the indirect effect of the alternative pathway was smaller than that of our hypothesized mediation model, and the percentage of indirect effect to total effect in the alternative model was also smaller than that of the hypothesized mediation model (see online Supplementary Materials). These findings suggest that our hypothesized mediation model is more appropriate than the alternative model to characterize the data. We present results pertaining to our hypothesized model.

## 2. Study 1

Study 1 tested the negative association between the perceived risk of COVID-19 pandemic and sense of control at the individual level.

### 2.1. Methods

#### 2.1.1. Participants

According to Schönbrodt and Perugini [67], a sample of 250 would be recommended for stable estimates of bivariate correlations. We recruited 265 participants via the Chinese online platform Credamo. Five were excluded for failing to complete the survey, which left 260 participants in the final sample (104 men, 156 women; *M*_age_ = 28.68, *SD*_age_ = 6.09). Participants provided informed consent and received CNY 2 for their participation.

#### 2.1.2. Procedures and Materials

Participants completed a set of questionnaires that assessed their perceived risk of the COVID-19 pandemic and sense of control. The perceived risk of COVID-19 was measured by the 9-item COVID-19 Risk Perception Scale [68]. Participants rated their agreement with each item (e.g., “I think COVID-19 is very difficult to cure,” and “The pandemic is far from over, and there is a risk of infection at any time”) on a 5-point scale from 1 (*not at all*) to 5 (*extremely*). The scale had a Cronbach’s α = 0.86 in the current sample. The sense of control was measured by the 16-item General Sense of Control Subscale of the Shapiro Control Inventory [69]. Participants rated each item (e.g., “I can make choices and decisions about important things in my life,” and “There is a positive sense of control in my life”) on a 7-point scale from 1 (*never*) to 7 (*always*). The scale had a Cronbach’s α = 0.91 in the current sample. Finally, participants reported their demographic information and were debriefed and thanked. The procedure took approximately 5 min.

### 2.2. Results

As hypothesized, the perceived COVID-19 risk (*M* = 2.81, *SD* = 0.73) was negatively associated with sense of control (*M* = 5.29, *SD* = 0.75), *r* (260) = −0.21, *p* < 0.001. The partial correlation between risk perception and sense of control, controlling for age and gender, was −0.20. Thus, consistent with our hypothesis and previous observations [37,38], participants who perceived greater risks from COVID-19 exhibited a lower sense of control. 

## 3. Study 2

In Study 2, we manipulated the level of perceived risk of COVID-19 to test the causal relation between COVID-19 risk perception and sense of control.

### 3.1. Methods

#### 3.1.1. Participants

Findings from Study 1 showed a weak to moderate correlation between pandemic risk perception and sense of control, equivalent to Cohen’s *d* = 0.43. A G*Power analysis [70] showed that at least 172 participants would be needed to detect an effect size *d* = 0.43 for a between-subjects design with a power of 0.80 and α = 0.05. In anticipation of attrition [71], we recruited 213 participants via the Chinese online platform Credamo. We excluded 19 who failed to complete the measures and another 14 (see detail below) who did not pass the attention check. There were therefore 180 participants in the final sample (75 men, 105 women; *M*_age_ = 30.79, *SD*_age_ = 6.52). Participants provided informed consent and received CNY 3 for their participation. 

#### 3.1.2. Procedures and Materials

Participants were randomly assigned to a high risk-perception (*n* = 90) or low risk-perception condition (*n* = 90). We manipulated the level of COVID-19 risk perception using reading materials. Specifically, we presented participants in the high risk-perception condition with one text passage that reported a set of the latest data on COVID-19 that were highly negative (e.g., the number of confirmed cases and deaths). In contrast, we presented participants in the low risk-perception condition with one text passage that reported a set of the latest data on COVID-19 that were positive (e.g., the number of cured cases and discharges from hospitals). The text passages were approximately 350 words in length and were both downloaded from the official website of the National Health Commission of the People’s Republic of China (http://www.nhc.gov.cn/ accessed on 9 December 2021). 

Participants were instructed to read the text carefully and to answer 4 reading comprehension questions that were designed to ensure that they had paid close attention to the reading material. Participants (*n* = 14, see above) who failed to correctly answer all 4 questions were excluded from further analysis. Afterwards, for a manipulation check, participants completed the 9-item COVID-19 Risk Perception Scale [68], which had a Cronbach’s α = 0.86. Finally, we assessed participants’ sense of control with the 12-item Sense of Control Scale [72]. Participants rated the items (e.g., “I can do just about anything I really set my mind to,” and “Whether or not I am able to get what I want is in my own hands”) on 7-point scales from 1 (*strongly disagree*) to 7 (*strongly agree*). The scale had a Cronbach’s α = 0.92 in the current sample. Finally, participants reported their demographic information and were debriefed and thanked. The entire procedure took approximately 5 min.

### 3.2. Results 

#### 3.2.1. Manipulation Check 

Participants in the high risk-perception condition (*M* = 3.04, *SD* = 0.65) perceived greater risk related to COVID-19 than those in the low risk-perception condition (*M* = 2.48, *SD* = 0.65), *t* (178) = 5.78, *p* < 0.001, *d* = −0.86, 95% CI = [0.37, 0.75]. The manipulation was thus effective. The two groups did not differ significantly in age (*p* = 0.42) or gender (*p* = 0.05) composition.

#### 3.2.2. Risk Perception and Sense of Control

As predicted, participants in the high risk-perception condition (*M* = 5.02, *SD* = 1.17) reported lower levels of sense of control than those in the low risk-perception condition (*M* = 5.44, *SD* = 0.75), *t* (151.90) = −2.87, *p* = 0.005, *d* = 0.43, and 95% CI = [−0.71, −0.13]. These findings provide experimental evidence that pandemic risk perception decreased individuals’ sense of control. They extend the correlational results in Study 1 and previous research [37,38]. 

## 4. Study 3a

Study 3a tested the hypothesis that death anxiety would mediate the effect of COVID-19 risk perception on sense of control.

### 4.1. Methods

#### 4.1.1. Participants

We used the Monte Carlo Power Analysis for Indirect Effects application [73] to determine the sample size for our proposed mediation model. As pandemic risk perception has a medium-sized effect on death anxiety [42] and sense of control (Studies 1 and 2), we applied a medium effect size in the power analysis. It was estimated that at least 150 participants were needed to reach a power of 0.80, with intercorrelations at *r* = 0.30 (*SD* = 1.00). We recruited 311 participants via the Chinese online platform Credamo, and 4 were excluded for failing the attention check. The final sample included 307 participants (114 men, 193 women; *M*_age_ = 30.77, *SD*_age_ = 13.35). Participants provided informed consent and received CNY 3for their participation. 

#### 4.1.2. Procedures and Materials

Participants were randomly assigned to a high risk-perception (*n* = 155) or low risk-perception condition (*n* = 152). We manipulated the level of risk perception by asking participants to watch a video. We downloaded the latest data on COVID-19 from the official website of the National Health Commission of the PRC (http://www.nhc.gov.cn/ accessed on 20 December 2021) and compiled two text passages with negative and positive data, respectively, each in about 350 words. Then, we imported the two text passages into a video-editing app to create two videos with COVID-19 images and sounds similar to news broadcasts, each approximately 90 s long. The video containing mainly negative information related to COVID-19 was presented to participants in the high risk-perception condition, whereas the video containing mainly positive information related to COVID-19 was presented to participants in the low risk-perception condition. 

Participants were instructed to watch the video carefully and answer a comprehension question. Then, for a manipulation check, participants completed 3 items about COVID-19 risk perception [28] (e.g., “No matter how small the odds, I could be infected with the novel Coronavirus”), rating on 6-point scales from 1 (*strongly disagree*) to 6 (*strongly agree*) (Cronbach’s α = 0.75). Next, they completed the 15-item Death Anxiety Scale [39], where they rated the items (e.g., “I am very afraid to die” and “I fear dying a painful death”) as 1 = *yes* or 0 = *no* (Cronbach’s α = 0.90). Finally, participants completed the Sense of Control Scale, the same as in Study 2 [72] (Cronbach’s α = 0.91). Participants then reported their demographic information and were debriefed and thanked. The two groups did not differ significantly in age (*p* = 0.42) or gender (*p* = 0.05) composition.

### 4.2. Results 

#### 4.2.1. Manipulation Check

Participants in the high risk-perception condition (*M* = 3.79, *SD* = 0.92) perceived more risk from COVID-19 than those in the low risk-perception condition (*M* = 2.96, *SD* = 0.84), *t* (305) = 8.28, *p* < 0.001, *d* = −0.95, 95% CI = [0.63, 1.03]. The manipulation was thus effective. The two groups did not differ significantly in age (*p* = 0.93) or gender (*p* = 0.40) composition.

#### 4.2.2. The Role of Death Anxiety in Risk Perception and Sense of Control

A one-way between-group multivariate analysis of variance (MANOVA) was performed to investigate the condition effect on death anxiety and sense of control. The results showed that participants in the high risk-perception condition (*M* = 0.75, *SD* = 0.25) reported a higher level of death anxiety than those in the low risk-perception condition (*M* = 0.57, *SD* = 0.30), *F* (1, 305) = 30.67, *p* < 0.001, η*_p_*^2^ = 0.09. Furthermore, participants in the high risk-perception condition (*M* = 4.58, *SD* = 1.08) reported a lower level of sense of control than their counterparts (*M* = 5.03, *SD* = 0.91), *F* (1, 305) = 16.19, *p* < 0.001, η*_p_*^2^ = 0.05.

**Figure 1:**
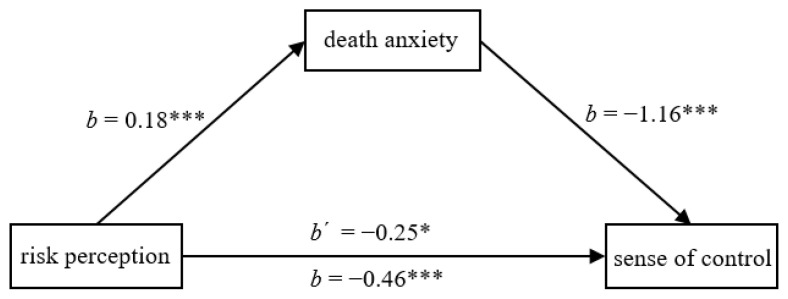
Death anxiety partially mediated the effect of risk perception on sense of control in Study 3a. * *p* < 0.05, *** *p* < 0.001.

We then performed mediation analysis. We entered the risk-perception condition as the predictor (1 = *low risk perception*, 2 = *high risk perception*), death anxiety as the mediator, and sense of control as the outcome. The indirect effect was significant, *b* = −0.20, *SE* = 0.05, 95% CI [−0.32, −0.12] (see Figure 1). Death anxiety thus partially mediated the effect of COVID-19 risk perception on sense of control.

Pirlott and MacKinnon [74] suggested a double randomization design as an effective means of verifying the mediation model in experimental designs. This design includes Step 1 of randomly assigning participants to manipulated X (predictor) and measuring M (mediator) and Y (outcome), and Step 2 of randomly assigning participants to manipulated M and measuring Y. Study 3a completed Step 1. Next, we carried out Step 2 in Study 3b to provide more convincing evidence for our mediation model.

## 5. Study 3b

In Study 3b, we manipulated the level of death anxiety to provide further evidence for the mediating role of death anxiety (M) in the effect of COVID-19 risk perception (X) on sense of control (Y). We expected that death anxiety would have a negative impact on sense of control.

### 5.1. Methods

#### 5.1.1. Participants

Given that death anxiety was moderately correlated with sense of control in Study 3a (*r* = −0.37), we applied a medium effect size in a power analysis to determine the sample size. A G*Power analysis [70] showed that at least 128 participants were needed to detect a medium effect size of *d* = 0.5 for a between-subjects design with a power of 0.80 and α = 0.05. We recruited 178 participants via the Chinese online platform Credamo, and 17 were excluded for not passing the attention check. The final sample thus included 161 participants (46 men, 114 women; *M*_age_ = 28.99, *SD*_age_ = 8.09). Participants provided informed consent and received CNY 10 for their participation. 

#### 5.1.2. Procedures and Materials

Participants were randomly assigned to a death anxiety (*n* = 85) or negative emotion condition (*n* = 76). We manipulated the level of death anxiety using the mortality salience paradigm [75]. First, participants in both conditions were asked to complete the 10-item Big Five Personality Inventory, which was intended to conceal the true purpose of the study. Then, participants in the death anxiety condition were presented with a short essay of approximately 500 words that described in first person how a main character noticed a lump in his/her abdomen and was eventually diagnosed with colorectal cancer. Participants in the negative emotion condition were presented with an essay of similar length that described how a main character noticed his/her decayed teeth and eventually had a dental surgery. In both conditions, participants were instructed to read the essay carefully and experience the mood of the protagonist as much as possible, and then to answer 3 reading comprehension questions. 

Greenberg et al. [76] have demonstrated that a distraction task after a mortality salience manipulation is necessary because the accessibility to death-related thoughts does not increase immediately but after a delay. Accordingly, after the mortality salience manipulation, we asked participants to complete a distraction task, in which they completed the 20-item Positive and Negative Affect Scale (PANAS) [77] and the 40-item Chinese version of Positive and Negative Affect Scale-Expanded (PANAS-X) [78]. Then, participants completed the same scale for sense of control as in Study 2 [72] (Cronbach’s α = 0.87), followed by the 15-item Death Anxiety Scale [39] (Cronbach’s α = 0.86) for a manipulation check. Finally, participants reported their demographic information and were debriefed and thanked. The entire procedure took approximately 8 min.

### 5.2. Results 

#### 5.2.1. Manipulation Check

Participants in the death anxiety condition (*M* = 0.71, *SD* = 0.23) reported a significantly higher level of death anxiety than those in the negative emotion condition (*M* = 0.56, *SD* = 0.27), *t* (159) = 3.76, *p* < 0.001, *d* = −0.59, 95% CI = [0.07, 0.23]. Moreover, participants in the death anxiety condition also reported more negative affect (*M* = 2.34, *SD* = 0.81), *t* (159) = 1.97, *p* = 0.05, *d* = −0.31, 95% CI = [−0.00, 0.50], and less positive affect (*M* = 2.75, *SD* = 0.92), *t* (159) = −2.87, *p* = 0.005, *d* = 0.45, 95% CI = [−0.70, −0.13], than those in the negative emotion condition (negative affect *M* = 2.09, *SD* = 0.80; positive affect *M* = 3.16, *SD* = 0.90). Controlling for positive and negative affect, the difference in death anxiety between the two conditions remained significant, *F* (1, 157) = 8.55, *p* = 0.004, η*_p_*^2^ = 0.05. The manipulation was thus effective. The two groups did not differ significantly in age (*p* = 0.09) or gender (*p* = 0.94) composition.

#### 5.2.2. Death Anxiety and Sense of Control

As predicted, participants in the death anxiety condition (*M* = 4.56, *SD* = 0.92) reported a lower level of sense of control than those in the negative emotion condition (*M* = 4.95, *SD* = 0.83), *t* (159) = −2.79, *p* = 0.006, *d* = 0.44, 95% CI = [−0.66, −0.11], indicating that death anxiety decreased sense of control. These findings provide additional evidence for the mediating role of death anxiety in the effect of COVID-19 risk perception on sense of control. 

## 6. Study 4

Study 4 further investigated the boundary condition of the effect of COVID-19 risk perception on death anxiety and sense of control by taking into account culturally informed coping. Confucian coping, a coping style manifesting prominent Confucian characteristics, includes three dimensions: fate thinking, pro-setback thinking, and responsibility thinking [59]. Research has suggested that pro-setback thinking reflects cognitive re-framing and is a particularly effective coping strategy [65,66]. In the COVID-19 context, pro-setback thinking may be the most helpful to mitigate the negative impact of pandemic risk perception. Thus, we focused on the dimension of pro-setback thinking in Study 4 to examine the role of Confucian coping in moderating the effect of COVID-19 risk perception on sense of control.

### 6.1. Methods

#### 6.1.1. Participants and Design

A G*Power [70] analysis showed that at least 128 participants were needed to detect a medium effect size of *f* = 0.25 for a 2 × 2 between-subjects design with a power of 0.80 and α = 0.05. We recruited 460 participants via the Chinese online platform Credamo. One participant was excluded for not passing the attention check. The final sample included 459 participants (171 men, 286 women, and two people reported other gender; *M*_age_ = 27.65, *SD*_age_ = 8.46). Participants provided informed consent and received CNY 3 for their participation.

#### 6.1.2. Procedure and Materials

Participants were randomly assigned to one of the four conditions in a 2 (risk perception: high (*n* = 227) vs. low (*n* = 232)) × 2 (Confucian coping: pro-setback (*n* = 234) vs. control (*n* = 225)) between-subjects design. We manipulated risk perception similarly to Study 2, except that a memory task was added to reinforce the manipulation. After reading the text passage about the latest pandemic data (accessed on 20 January 2022), participants were asked to recall and describe in about 50 words their positive (for the low-risk condition) or negative (for the high-risk condition) experiences during the COVID-19 pandemic. Then, for a manipulation check, they responded to 3 items about the perception of COVID-19 risk [28] (Cronbach’s α = 0.74), the same as in Study 3a.

Next, the Confucian-coping manipulation was carried out. Participants in the pro-setback condition were asked to read a 500-word text passage that described the importance of facing and overcoming setbacks and remaining positive according to Confucian teaching, with examples of famous individuals in Chinese history who actively overcame setbacks and obtained great achievements. Participants were then instructed to reflect on their lives and describe in about 100 words a memorable incident in which they or someone close to them overcame a setback. Participants in the control condition read a text passage of a similar length that described the workday routine of a person named Lin. They were then asked to reflect on their lives and describe in about 100 words their routine on weekdays. For a manipulation check, participants in both conditions completed 4 items on pro-setback thinking in Confucian coping [65]. They rated the items (e.g., “I often think that only those who have suffered many setbacks can achieve great things”) on 5-point scales from 1 (*strongly disagree*) to 5 (*strongly agree*), which had a Cronbach’s α = 0.68. 

Then, participants completed the 15-item Death Anxiety Scale [39], as in Study 3a. To effectively assess individual variation, we employed a 7-point Likert scale to replace the original “yes/no” scoring [47] (1 = *strongly disagree*, 7 = *strongly agree*; Cronbach’s α = 0.87). Finally, participants completed the 3-item Sense of Control Scale [51], where they rated the items (e.g., “At the moment, I feel a lack of control” reverse scored) on 7-point scales (1 = *strongly disagree*, 7 = *strongly agree*; Cronbach’s α = 0.87). Participants then reported their demographic information and were debriefed and thanked. The entire procedure took approximately 10 min.

### 6.2. Results

#### 6.2.1. Manipulation Check 

Participants in the high risk-perception condition (*M* = 3.60, *SD* = 0.95) perceived more risk from COVID-19 than those in the low risk-perception condition (*M* = 3.00, *SD* = 0.93), *t* (457) = 6.83, *p* < 0.001, *d* = −0.64, 95% CI = [0.43, 0.77]. The pandemic risk-perception manipulation was thus effective. Furthermore, participants in the pro-setback condition (*M* = 3.65, *SD* = 0.66) reported a higher level of pro-setback thinking than those in the control condition (*M* = 3.29, *SD* = 0.72), *t* (457) = 5.58, *p* < 0.001, *d* = −0.52, 95% CI = [0.23, 0.48]. The Confucian coping manipulation was thus effective. The two risk-perception groups did not differ significantly in age (*p* = 0.67) or gender (*p* = 0.15) composition. The two coping groups also did not differ significantly in age (*p* = 0.07) or gender (*p* = 0.98) composition.

#### 6.2.2. Death Anxiety and Sense of Control

**Figure 2:**
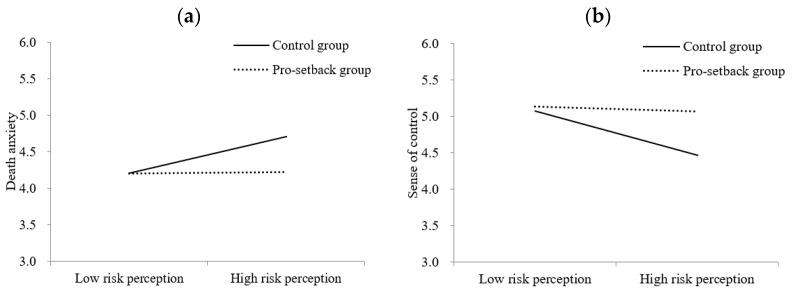
Confucian coping moderated the effect of risk perception on death anxiety (**a**) and sense of control (**b**) in Study 4.

We carried out a 2 (risk perception: high vs. low) × 2 (Confucian coping: pro-setback vs. control) ANOVA on death anxiety. There were significant main effects of risk perception, *F* (1, 455) = 8.40, *p* = 0.004, η*_p_*^2^ = 0.018, and Confucian coping, *F* (1, 455) = 7.85, *p* = 0.005, η*_p_*^2^ = 0.017, qualified by a risk-perception × Confucian-coping interaction, *F* (1, 455) = 7.62, *p* = 0.006, η*_p_*^2^ = 0.016. In the pro-setback condition, there was no significant difference in death anxiety between participants in the high (*M* = 4.22, *SD* = 1.00) and low risk-perception conditions (*M* = 4.21, *SD* = 0.99), *F* (1, 232) = 0.009, *p* = 0.925, η*_p_*^2^ = 0.000. In contrast, in the control condition, participants in the high risk-perception condition (*M* = 4.71, *SD* = 0.81) experienced greater death anxiety than did those in the low risk-perception condition (*M* = 4.21, *SD* = 0.96), *F* (1, 223) = 17.71, *p* < 0.001, η*_p_*^2^ = 0.074. Confucian coping thus moderated the effect of COVID-19 risk perception on death anxiety (see Figure 2a).

Next, we conducted a similar 2 × 2 ANOVA on sense of control. There were significant main effects of risk perception, *F* (1, 455) = 7.53, *p* = 0.006, η*_p_*^2^ = 0.016, and Confucian coping, *F* (1, 455) = 6.98, *p* = 0.009, η*_p_*^2^ = 0.015, qualified by a risk perception × Confucian coping interaction, *F* (1, 455) = 4.68, *p* = 0.031, η*_p_*^2^ = 0.010. Whereas in the pro-setback condition, there was no significant difference in sense of control between participants in the high (*M* = 5.06, *SD* = 1.23) and low risk-perception conditions (*M* = 5.14, *SD* = 1.41), *F* (1, 232) = 0.175, *p* = 0.676, η*_p_*^2^ = 0.001, in the control condition, participants in the high risk-perception condition (*M* = 4.47, *SD* = 1.43) scored lower on sense of control than did those in the low risk-perception condition (*M* = 5.08, *SD* = 1.25), *F* (1, 223) = 11.64, *p* = 0.001, η*_p_*^2^ = 0.050. Thus, Confucian coping moderated the effect of COVID-19 risk perception on sense of control (see Figure 2b).

#### 6.2.3. Mediation and Moderated Mediation Analysis

**Figure 3:**
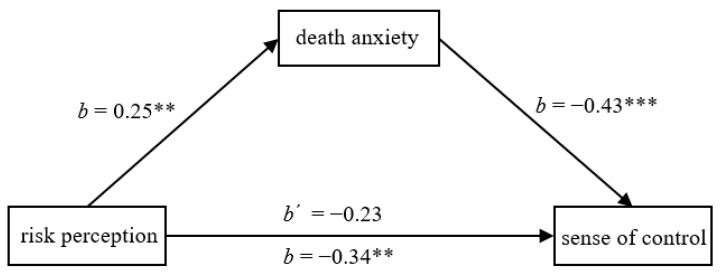
Death anxiety mediated the effect of risk perception on sense of control in Study 4. ** *p* < 0.01, *** *p* < 0.001.

We further conducted a mediation analysis, entering the risk-perception condition as the predictor (1 = *low risk perception*, 2 = *high risk perception*), death anxiety as the mediator, and sense of control as the outcome. The indirect effect was significant, *b* = −0.11, *SE* = 0.04, 95% CI [−0.20, −0.03] (see Figure 3). This replicated the mediation findings of Study 3a.

**Figure 4:**
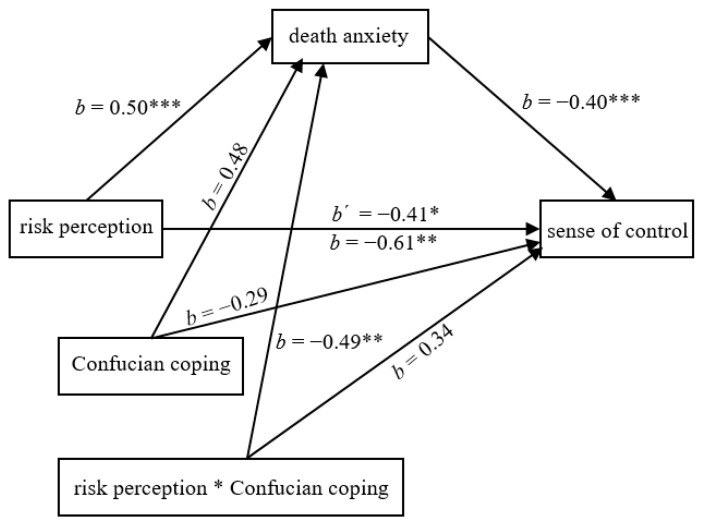
The Moderated Mediation Model in Study 4. * *p* < 0.05, ** *p* < 0.01, *** *p* < 0.001.

Next, we examined the moderating role of the Confucian coping strategy of pro-setback thinking in a moderated mediation model. We entered risk perception as the predictor (1 = *the low risk perception*, 2 = *the high risk perception*), death anxiety as the mediator, sense of control as the outcome, and Confucian coping (1 = *pro-setback condition*, 0 = *control condition*) as the moderator into Model 8 [79] (PROCESS 2.16.3; 5000 iterations). The risk-perception × Confucian-coping interaction effect on death anxiety was significant, *b* = −0.49, *SE* = 0.18, 95% CI [−0.83, −0.14], whereas the risk-perception × Confucian-coping interaction effect on sense of control was not significant, *b* = 0.34, *SE* = 0.24, 95% CI [−0.13, 0.82], with moderated mediation index = 0.20, *SE* = 0.08, 95% CI [0.06, 0.36]. These results suggest that Confucian coping moderated the mediating role of death anxiety (see Figure 4). In the pro-setback condition, the mediating effect of death anxiety was not significant, *b* = −0.01, *SE* = 0.05, 95% CI [−0.11, 0.10]. In contrast, in the control condition, the mediating effect of death anxiety was significant, *b* = −0.20, *SE* = 0.06, 95% CI [−0.34, −0.10]. Thus, Confucian coping reduced death anxiety that was triggered by COVID-19 risk perception and indirectly mitigated the negative effect of risk perception on sense of control.

## 7. Study 5

To further test our hypotheses in a real-world context [80], Study 5 employed a longitudinal design with three waves of data collection. In addition, extending Study 4, that only examined the protective role of pro-setback thinking, Study 5 tested all three dimensions of Confucian coping as effective means to alleviate the negative impact of COVID-19 risk perception on sense of control.

### 7.1. Methods

#### 7.1.1. Participants

We collected data in three waves via the Chinese online platform Credamo, with 1164 participants in wave 1, 891 remaining in wave 2, and 835 remaining in wave 3. Thus, the final sample included 835 participants who completed all three waves of data collection (329 men, 506 women; *M*_age_ = 30.49, *SD*_age_ = 7.37). Participants provided informed consent and received CNY 6 for their participation. Participants who left the study did not differ in gender composition from those who completed the study (*p* = 0.80), but they (*M*_age_ = 27.89, *SD*_age_ = 5.77) were significantly younger (*p* < 0.001).

#### 7.1.2. Procedures and Materials

Data collection commenced in January 2022 on Credamo. In wave 1, participants completed the Risk Perception Scale [68] and Confucian Coping Scale [65] and provided demographic information. The procedure took less than 5 min. Wave 2, in which participants completed the Death Anxiety Scale [39], followed 2 weeks later. The procedure took less than 3 min. Then, 4 weeks after wave 1, wave 3 data collection was carried out, in which participants completed the Sense of Control Scale [69]. The procedure took less than 3 min.

*Perceived risk from the COVID-19 pandemic*. We used the same scale to measure COVID-19 risk perception as in Study 1 [68] (Cronbach’s α = 0.83).

*Confucian coping*. The 12-item Confucian Coping Scale was used [65]. The scale includes 3 subscales: the fate-thinking subscale (e.g., “Whether an event ends well or bad is predetermined by fate;” Cronbach’s α = 0.88), the pro-setback-thinking subscale (the same as in Study 4, Cronbach’s α = 0.76), and the responsibility-thinking subscale (e.g., “When frustrated, I still do well what I should do;” Cronbach’s α = 0.71), each of which contains 4 items. The three subscales of Confucian coping were analyzed separately. Participants rated the items on 5-point scales (1 = *strongly disagree*, 5 = *strongly agree*). 

*Death anxiety*. The 15-item Death Anxiety Scale [39] was used. As in Study 4, we employed a 7-point Likert scale to capture individual variation [47] (1 = *strongly disagree*, 7 = *strongly agree*; Cronbach’s α = 0.94).

*Sense of control*. The same scale for sense of control as in Study 1 was used [69] (Cronbach’s α = 0.93).

### 7.2. Results

#### 7.2.1. Test of Common Method Variance

The common method bias was tested according to the Harman’s single-factor test [81]. The variance interpretation rate of the first factor was 25.49%, lower than the standard of 40%, which indicates that there was no obvious common method bias in the data.

#### 7.2.2. Mediation Analysis

**Figure 5:**
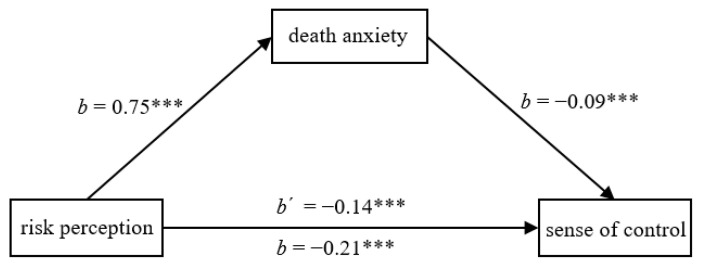
Death anxiety partially mediated the effect of risk perception on sense of control in Study 5. *** *p* < 0.001.

We entered the perceived risk from the COVID-19 pandemic as the predictor, death anxiety as the mediator, sense of control as the outcome, and gender and age as covariates in a mediation model. The indirect effect was significant, *b* = −0.07, *SE* = 0.02, 95% CI [−0.11, −0.04] (see Figure 5). Thus, death anxiety partially mediated the effect of risk perception at an earlier time point on sense of control 4 weeks later. 

#### 7.2.3. Moderated Mediation Analysis

We then conducted three moderated mediation models with pro-setback thinking, fate thinking, and responsibility thinking as the moderators, respectively. Across all models, we entered the perceived risk of COVID-19 as the predictor, death anxiety as the mediator, sense of control as the outcome, the Confucian coping dimension as the moderator, and gender and age as covariates into Model 8 [79] (PROCESS 2.16.3; 5000 iterations). 

**Figure 6:**
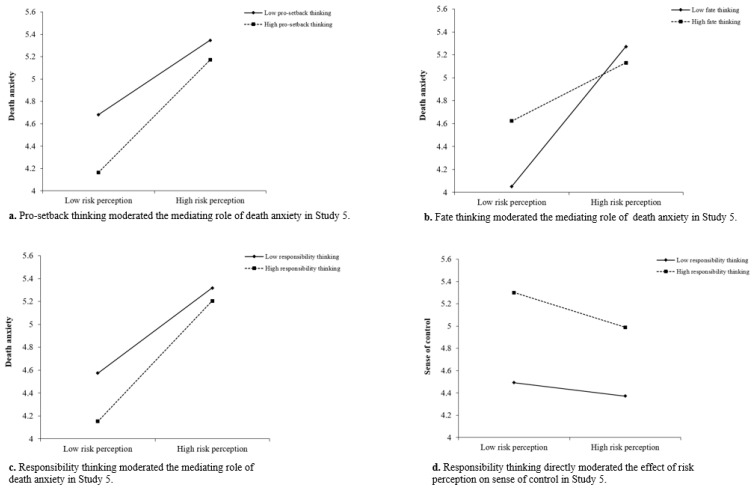
Confucian coping moderated the effect of risk perception on sense of control and the mediating role of death anxiety in Study 5.

In the model for pro-setback thinking, the risk-perception × pro-setback-thinking interaction on death anxiety was significant, *b* = 0.17, *SE* = 0.07, 95% CI [0.03, 0.31], whereas the risk-perception × pro-setback-thinking interaction on sense of control was not significant, *b* = −0.002, *SE* = 0.04, 95% CI [−0.08, 0.08], with moderated mediation index = −0.01, *SE* = 0.01, 95% CI [−0.026, −0.001]. This suggests that pro-setback thinking moderated the mediating role of death anxiety. By dividing pro-setback thinking scores into high and low groups according to the mean plus/minus 1 standard deviation, the simple slope test (see Figure 6a) showed that there was a significant indirect effect of risk perception on sense of control via death anxiety at the low level of pro-setback thinking, *b* = −0.03, *SE* = 0.01, 95% CI [−0.06, −0.01], as well as at the high level of pro-setback thinking, *b* = −0.05, *SE* = 0.02, 95% CI [−0.09, −0.01]. Importantly, death anxiety was lower among those with a high level of pro-setback thinking than those with a low level of pro-setback thinking, regardless of the level of COVID-19 risk perception. 

Similarly, in the model for fate thinking, the risk-perception × fate-thinking interaction on death anxiety was significant, *b* = −0.34, *SE* = 0.06, 95% CI [−0.46, −0.21], while the interaction was not significant for sense of control, *b* = 0.03, *SE* = 0.04, 95% CI [−0.04, 0.10], with moderated mediation index = 0.03, *SE* = 0.01, 95% CI [0.01, 0.04]. This suggests that fate thinking moderated the mediating role of death anxiety. By dividing fate thinking scores into high and low groups according to the mean plus/minus 1 standard deviation, the simple slope test (see Figure 6b) showed that there was a significant indirect effect of risk perception on sense of control via death anxiety at the low level of fate thinking, *b* = −0.07, *SE* = 0.02, 95% CI [−0.12, −0.04], as well as the high level of fate thinking, *b* = −0.03, *SE* = 0.01, 95% CI [−0.06, −0.01]. Death anxiety was lower among those with a high level of fate thinking than those with a low level of fate thinking when the level of COVID-19 risk perception was high, but the pattern was reversed when the level of COVID-19 risk perception was low. Thus, it appears that fate thinking contributed to decreased death anxiety and indirectly protected the sense of control when the risk of COVID-19 was perceived as high.

Finally, in the model for responsibility thinking, the risk-perception × responsibility-thinking interaction on death anxiety was significant, *b* = 0.28, *SE* = 0.12, 95% CI [0.05, 0.51], and the interaction effect on sense of control was also significant, *b* = −0.16, *SE* = 0.06, 95% CI [−0.28, −0.04], with moderated mediation index = −0.02, *SE* = 0.01, 95% CI [−0.038, −0.002]. This suggests that responsibility thinking not only moderated the mediating role of death anxiety, but also directly moderated the effect of risk perception on sense of control. By dividing responsibility thinking scores into high and low groups according to the mean plus/minus 1 standard deviation, the simple slope test showed that there was a significant indirect effect of risk perception on sense of control via death anxiety at the low level of responsibility thinking, *b* = −0.03, *SE* = 0.01, 95% CI [−0.06, −0.01], as well as at the high level of responsibility thinking, *b* = −0.05, *SE* = 0.02, 95% CI [−0.09, −0.02] (see Figure 6c). Furthermore, there was a significant direct effect of risk perception on sense of control at the high level of responsibility thinking, *b* = −0.20, *SE* = 0.04, 95% CI [−0.28, −0.12], but not at the low level of responsibility thinking, *b* = −0.06, *SE* = 0.05, 95% CI [−0.15, 0.03] (see Figure 6d). Importantly, death anxiety was lower and sense of control was higher among those with a high level of responsibility thinking than those with a low level of responsibility thinking, regardless of the level of COVID-19 risk perception.

Taken together, all three dimensions of Confucian coping helped to reduce death anxiety and further prevent sense of control from declining. This remained true among participants with both low and high risk perceptions, expect for fate thinking, where, when COVID-19 was perceived as low risk, death anxiety was actually higher among those with high fate thinking than among those with low fate thinking. Interestingly, the differences between participants with low versus high Confucian thinking appeared larger among those with low risk-perceptions than those with high risk-perceptions in the current sample. We are hesitant to speculate, especially in light of the findings of Study 4 where differences between the Confucian thinking versus control groups were not significant in the low-risk condition but significant in the high-risk condition. Future research should examine how people with low versus high Confucian thinking respond when transitioning from low-risk to high-risk situations like the COVID-19 crisis.

## 8. General Discussion

The present research yielded original findings concerning the adverse impact of COVID-19 risk perception on individuals’ sense of control and the related contributing and mitigating factors. Across six studies that utilized different methods and research designs, we found that individuals’ risk perception of COVID-19 undermined their sense of control and that the effect was mediated by death anxiety. Furthermore, Confucian coping contributed to a decrease in death anxiety and further protected sense of control from decline because of COVID-19 risk perception. Given the critical impact of sense of control on mental and physical health [4,5,82], these findings have important real-life implications.

In extending existing findings on the negative association between risk perception and sense of control [37,38], the current findings established the causal effect of COVID-19 risk perception on individuals’ sense of control. Whereas prior research has shown that people’s sense of control decreases when they are confronted with an uncontrollable event [32,33,34], the current studies highlight the process of risk perception of the uncontrollable event, namely the COVID-19 pandemic [30,83], in causing sense of control to decline. The findings thus contribute to the understanding of the antecedent of changes in sense of control.

Furthermore, the present research revealed an underlying mechanism for the negative effect of COVID-19 risk perception on sense of control, namely, death anxiety. People experience a loss of control after perceiving the risk of COVID-19 partly because their anxiety over death is triggered by the perceived risk, which, in turn, leads to a reduced sense of control. These findings complement results from other studies [49,50,55] showing that due to the widespread nature of the COVID-19 pandemic and the increasing numbers of confirmed cases and deaths [28], people frequently experience death anxiety [42], which can further have negative implications for their sense of control [54,55].

Moreover, the current findings reveal Confucian coping as an effective means that protects individuals from death anxiety due to COVID-19 risk perception and helps them maintain a sense of control. When participants were reminded of Confucian thinking, high risk perception did not result in increased death anxiety or reduced sense of control. Furthermore, high levels of pro-setback and responsibility thinking mitigated COVID-related death anxiety and in turn protected sense of control. A high level of fate thinking helped to reduce death anxiety and protect sense of control when the COVID-19 risk was perceived to be high. These results highlight the importance of considering coping and other resilience factors in specific cultural contexts [57,58]. In particular, although fate thinking is often considered a negative or passive coping style in the Western cultural context, it is a commonly used strategy among Asian individuals to deal with stressors in life [61,62,64,66]. Attributing the pandemic to fate, viewing setbacks as a path to growth, and taking responsibility in times of challenges may allow individuals to maintain a positive state of mind, which in turn protects them from the adverse impact of COVID-19.

In spite of the original contributions, the current research has important limitations. First of all, data collection of the studies took place either at one point in time (Studies 1–4) or across a short time span (Study 5). The changing circumstances during the COVID-19 pandemic were therefore not taken into consideration. For example, risk perception might be different before and after the vaccine became widely available. Future research may consider following individuals throughout an adverse event, collecting data at multiple times with baseline measures, to further understand how societal, sociocultural, and individual factors influence risk perception and its psychological impacts. Furthermore, this research was conducted in the context of COVID-19, a global public health outbreak, although data collection took place in a period of very little mortality in China, which could have affected death anxiety and risk perception. In addition, the coping strategies individuals use may differ with the different levels of the actual threat. The findings may therefore not be generalizable to other risk situations. Future studies examining other high-risk situations would help to corroborate the findings. Finally, the current studies focused on the Chinese population, and we did not collect information about the participants’ income or education. The participants were recruited from an online platform and might not be representative of the Chinese population. There might also be unintended response biases associated with paid participation. The generalizability of the findings to the Chinese population and other cultural groups thus requires examination. Still, the current research goes beyond WEIRD (Western, Educated, Industrialized, Rich, and Democratic) populations and enriches theories and dastabases in psychological science [84,85]. The findings shed new light on the psychological impact of risk perception in times of crisis and further suggest pathways to interventions.

## Data Availability

All data and research materials are available at https://osf.io/r9us5/?view_only=46c2cd66e94f42d4bee4adc9976ce646 (accessed between 1 December 2021 and 9 February 2022).

## Supplementary Materials

The following supporting information can be downloaded at: https://www.mdpi.com/article/10.3390/ijerph20032299/s1, Figure S1: Death anxiety partially mediated the effect of risk perception on sense of control in Study 3a; Figure S2: Sense of control partially mediated the effect of risk perception on death anxiety in Study 3a; Figure S3: Death anxiety mediated the effect of risk perception on sense of control in Study 4; Figure S4: Sense of control partially mediated the effect of risk perception on death anxiety in Study 4. Figure S5: Death anxiety partially mediated the effect of risk perception on sense of control in Study 5; Figure S6: Sense of control partially mediated the effect of risk perception on death anxiety in Study 5. Table S1–S3: Comparison of Mediation Models.
