# Supplementary material for: How Does COVID-19 Risk Perception Affect Sense of Control? The Roles of Death Anxiety and Confucian Coping
Source: Int J Environ Res Public Health. 2023 Jan 28;20(3):2299. doi: 10.3390/ijerph20032299 (PMC9916306; doi:10.3390/ijerph20032299)
Supplement: Supplementary file 1 [file ijerph-20-02299-s001.zip › ijerph-2136515-supplementary.pdf]

Supplemental material

Results of the alternative mediation model

In our article, we hypothesized based on the literature that death anxiety would be a mediator for the relation between risk perception and sense of control. However, an alternative possibility would be that sense of control served as a mediator for the relation between risk perception and death anxiety, such that higher risk perception might induce a lowered sense of control, which might in turn boost death anxiety. We therefore conducted additional mediation analyses to test the alternative path in Studies 3a, 4 and 5. The results showed that although the alternative mediation path was significant, the indirect effect of the alternative path was smaller than that of our hypothesized mediation model, and the percentage of indirect effect to total effect in the alternative path was also smaller than that of the hypothesized mediation model. These findings suggest that our hypothesized mediation model is more appropriate than the alternative model to characterize the data. The results of the alternative mediation models in studies 3a, 4 and 5 are presented below.

Study 3a

Table S1. Comparison of Mediation Models.

Mediator	Effect Type	Effect Value	Boot SE	Boot 95% LLCI	Boot 95% ULCI	Effect Percentage
Death Anxiety	Total Effect	-0.46	0.11	-0.68	-0.23	100.00%
	Direct Effect	-0.25	0.11	-0.48	-0.03	55.60%
	Indirect Effect	-0.20	0.05	-0.32	-0.11	44.40%
Sense of Control	Total Effect	0.18	0.03	0.11	0.24	100.00%
(**Alternative**)	Direct Effect	0.13	0.03	0.07	0.19	76.56%
	Indirect Effect	0.04	0.01	0.02	0.07	23.44%

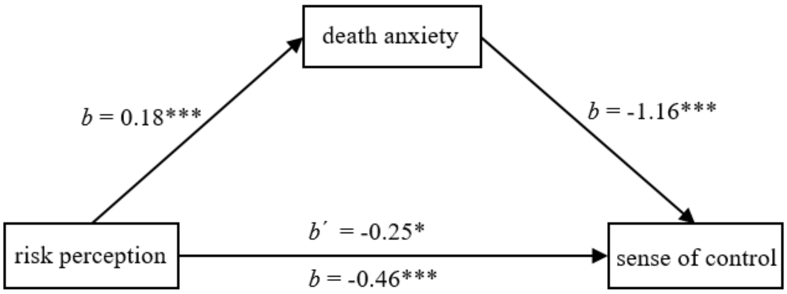

Figure S1. Death anxiety partially mediated the effect of risk perception on sense of control in Study 3a. * p <0.05, *** p < 0.001. (Hypothesized Mediation Model)

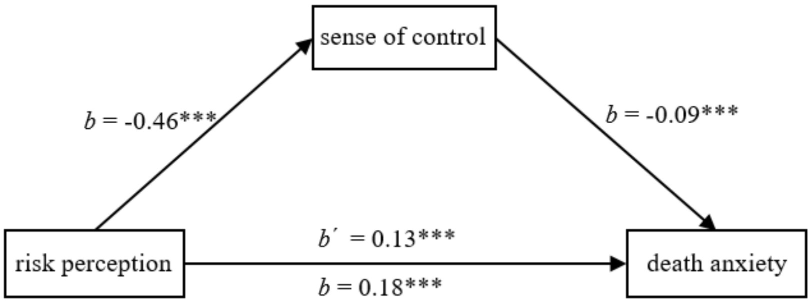

Figure S2. Sense of control partially mediated the effect of risk perception on death anxiety in Study 3a. *** $p < 0.001$. (Alternative Mediation Model)

Study 4

Table S2. Comparison of Mediation Models.

Mediator	Effect Type	Effect Value	Boot SE	Boot 95% LLCI	Boot 95% ULCI	Effect Percentage
Death Anxiety	Total Effect	-0.34	0.13	-0.58	-0.09	100.00%
	Direct Effect	-0.23	0.12	-0.47	0.01	67.65%
	Indirect Effect	-0.11	0.04	-0.20	-0.03	32.35%
Sense of Control	Total Effect	0.25	0.09	0.07	0.43	100.00%
(**Alternative**)	Direct Effect	0.18	0.09	0.01	0.35	72.00%
	Indirect Effect	0.07	0.03	0.02	0.14	28.00%

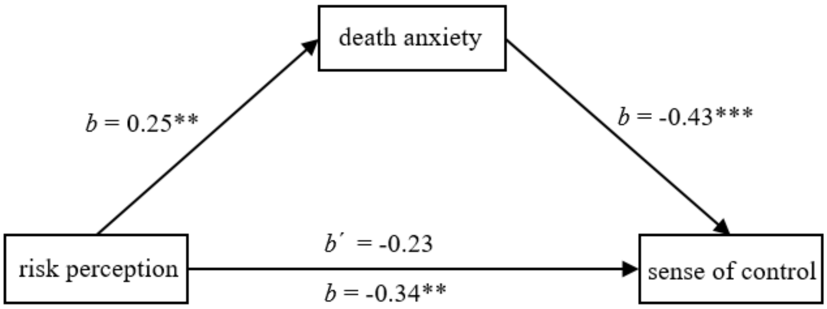

Figure S3. Death anxiety mediated the effect of risk perception on sense of control in Study 4. ** $p < 0.01$, *** $p < 0.001$. (Hypothesized Mediation Model)

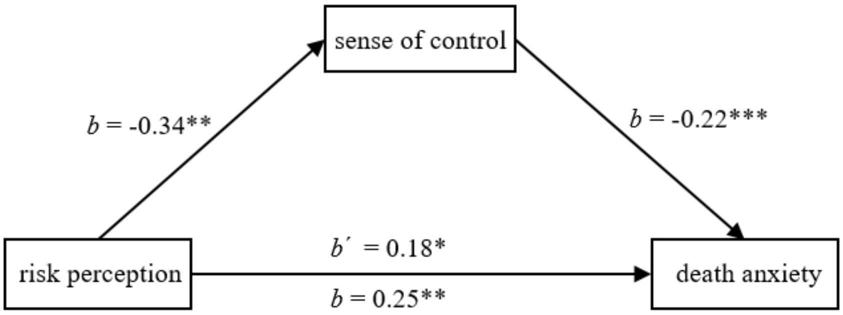

Figure S4. Sense of control partially mediated the effect of risk perception on death anxiety in Study 4. * $p < 0.05$, ** $p < 0.01$, *** $p < 0.001$. (Alternative Mediation Model)

Study 5

Table S3. Comparison of Mediation Models.

Mediator	Effect Type	Effect Value	Boot SE	Boot 95% LLCI	Boot 95% ULCI	Effect Percentage
Death Anxiety	Total Effect	-0.21	0.04	-0.29	-0.14	100.00%
	Direct Effect	-0.14	0.04	-0.22	-0.06	66.67%
	Indirect Effect	-0.07	0.02	-0.11	-0.04	33.33%
Sense of Control	Total Effect	0.75	0.06	0.63	0.87	100.00%
(**Alternative**)	Direct Effect	0.70	0.06	0.57	0.82	93.33%
	Indirect Effect	0.05	0.02	0.03	0.09	6.67%

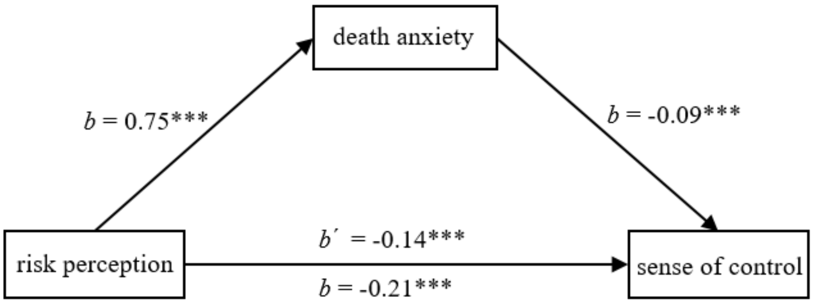

Figure S5. Death anxiety partially mediated the effect of risk perception on sense of control in Study 5. *** $p < 0.001$. (Hypothesized Mediation Model)

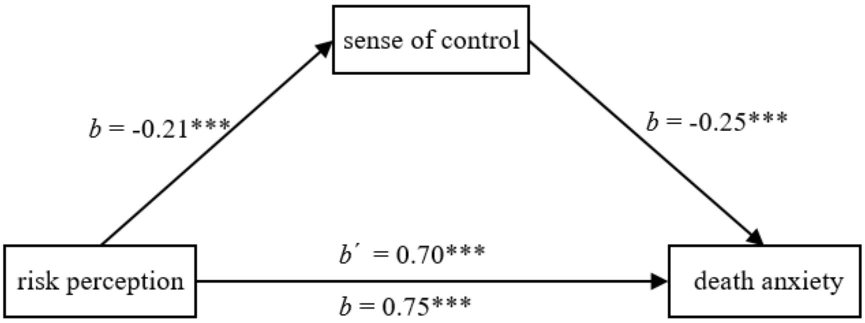

Figure S6. Sense of control partially mediated the effect of risk perception on death anxiety in Study 5. *** $p < 0.001$. (Alternative Mediation Model)
